# Older Adults and Family Discord or Violence During the COVID-19 Pandemic: Results of a Canada-Wide Survey

**DOI:** 10.1093/geroni/igab046.1060

**Published:** 2021-12-17

**Authors:** Gloria Gutman, Brian de Vries, Robert Beringer, Paneet Gill, Helena Dault, Mojgan Karbakhsh

**Affiliations:** 1 Simon Fraser University, Vancouver, British Columbia, Canada; 2 San Francisco State University, Palm Springs, California, United States; 3 University of Victoria, Victoria, British Columbia, Canada; 4 Simon Fraser University, Surrey, British Columbia, Canada; 5 Victoria Hospice, Victoria, British Columbia, Canada

## Abstract

Child abuse and intimate partner violence rates are known to increase during and in the aftermath of disasters. Research on elder abuse during disasters, including the current pandemic, is limited. As part of an online survey that explored older Canadians’ current experiences and future care plans during the COVID-19 pandemic, we aimed to determine the prevalence, contributing factors and potential outcomes of frequent family discord involving physical violence (FFD/PV) as a proxy for elder abuse. The survey was conducted between Aug 10 and Oct 10, 2021. Respondents (n=4380) were recruited using social media, direct email, Facebook advertising and with the assistance of 85 local community, regional and national organizations. The sub-sample reporting FFD/PV (n=76, 1.8%) was compared with other survey respondents regarding socio-demographic characteristics, negative and positive emotions, difficulty accessing basic needs, food, health care and support. Respondents experiencing FFD/PV were found to be significantly younger and less educated and were more likely to be non-white and not working than other respondents. The subgroup sustaining FFD/PV reported significantly higher rates of feeling depressed, lonely, isolated, anxious, sad, and judged/shamed and felt less happy, relaxed and accepted in their community. They also reported higher rates of challenges in accessing basic material needs such as food, support, medical care, mental health treatment and experienced more changes in life routines. Although only a small percentage reported FFD/PV, our results highlight a disturbing pattern that merits serious attention of adult protection agencies, seniors' advocates and disaster response organizations.

